# Improving Trust in Declaration-Based Exchange: Experimental Evidence on Competition and Contract Enforcement

**DOI:** 10.3390/bs16081356

**Published:** 2026-08-07

**Authors:** Siqi Wang, Wenkai Yang, Rui Wang, Yuli Ding, Bin Xu

**Affiliations:** School of Economics, Zhejiang Gongshang University, Hangzhou 310018, China; wangsiqi@mail.zjgsu.edu.cn (S.W.);

**Keywords:** trust, declaration, competition, contract enforcement

## Abstract

This paper studies how trust can be supported in exchange environments where potential responders make prior declarations about future return behavior. While prior work has separately examined communication, competition, or contract enforcement, how these mechanisms interact remains underexplored. We modify the standard trust game by introducing two potential responders who declare their intended returns before one is selected, before experimentally varying responder-side competition and contract enforcement in a 2 × 2 design. The results reveal an asymmetric relationship: competition significantly increases trust and realized trustworthiness, whereas contract enforcement alone does not generate a statistically significant increase in trust. However, when competition is combined with enforcement, the effect is substantially amplified, producing the highest levels of trust and trustworthiness. Mechanism evidence shows that competition drives higher declarations and selection incentives, while enforcement eliminates the declaration-fulfillment gap, making competitive declarations credible. These findings suggest that competition and enforcement are complements rather than substitutes—a result with implications for the design of online platforms and other markets where trust depends on prior declarations.

## 1. Introduction

The standard trust game (Berg et al., 1995) captures a fundamental problem in economic exchange: one party must decide whether to place resources under another party’s control before the other party’s future behavior is known. In the canonical setting, a sender decides how much of an initial endowment to transfer to a responder; the transferred amount is multiplied, after which the responder unilaterally decides how much to return. This structure mirrors many real-world transactions in which investment precedes performance, such as an investor providing seed capital based on projected returns, an online buyer paying an unfamiliar seller before receiving goods, or a lender evaluating a borrower’s nonbinding repayment statement. In such environments, trust is not formed in a vacuum. Potential trustees often communicate prior declarations about their intended future behavior, including advertised quality, return policies, delivery promises, repayment plans, or projected returns. These declarations may help trustors form expectations, but they also create a central behavioral problem: statements that attract trust ex ante may not be honored ex post.

This paper studies how trust can be supported in declaration-based exchange. We focus on two institutional mechanisms that are common in market transactions: responder-side competition and contract enforcement. These mechanisms target different dimensions of the exchange process. Competition operates through incentives. When multiple potential responders compete for the sender’s investment, they have stronger incentives to make attractive declarations in order to be selected. Contract enforcement, by contrast, operates through structural credibility: by making declared returns automatically binding, enforcement eliminates the execution gap between ex ante commitments and ex post realization. At first glance, both mechanisms appear capable of fostering trust. Yet they may operate asymmetrically. Competition changes the attractiveness of the sender’s choice environment, whereas enforcement mainly changes the credibility of already-made declarations. This distinction suggests that contract enforcement may not be a standalone generator of trust. Instead, enforcement may work primarily as a credibility amplifier: it strengthens the trust-enhancing effect of competition by transforming competitive declarations into binding commitments.

Prior research provides important insights into each part of this mechanism, but leaves their interaction underexplored. Studies on communication and promises show that nonbinding statements can promote cooperation by shaping beliefs, creating psychological costs of disappointing others, or activating guilt aversion (Charness & Dufwenberg, 2006; Schniter et al., 2013). However, the effect of such communication is fragile. Cheap talk and nonbinding promises may be informative, but they also allow trustees to engage in strategic overpromising, which can generate disappointment and erode subsequent trust when promises are not fulfilled (Bracht & Feltovich, 2009; Y. Chen & Zhang, 2021). A separate body of literature on market competition shows that partner selection can foster efficiency when buyers can rely on reputation, feedback, or information about past behavior (Bolton et al., 2008; Huck et al., 2012), while other forms of competition may weaken reciprocity, alter perceived trustworthiness, or impede reputation building (Keck & Karelaia, 2012; Bauernschuster et al., 2013; Hoffmann et al., 2021). A third literature on contracts and control suggests that formal enforcement can reduce discretion and opportunism, but may not automatically increase interpersonal trust and may sometimes substitute for, rather than create, trust (Malhotra & Murnighan, 2002; Bohnet et al., 2001; Falk & Kosfeld, 2006).

These findings point to a critical gap. Existing studies typically examine communication, competition, or enforcement separately, but many market exchanges combine all three: agents make declarations, compete for selection, and may or may not be held contractually accountable for what they declare. In such settings, the key question is not simply whether competition or enforcement increases trust, but how the two mechanisms interact. Does responder-side competition make declarations more informative, or does it merely induce more attractive but less credible claims? Does contract enforcement increase trust on its own, or does its main effect emerge only when it makes competitive declarations credible? Put differently, is enforcement a direct source of trust, or does it act primarily as an amplifier of competition?

To answer these questions, we design a 2 × 2 laboratory experiment based on a modified trust game. In each group, one sender faces two potential responders. Before matching is determined, responders declare how much they will return if selected. We vary two institutional dimensions. The first is responder-side competition: in the competition condition, the sender observes both declarations and chooses which responder to match with; in the no-competition condition, the responder is randomly selected. The second is contract enforcement: in the enforcement condition, the declared return is automatically implemented as the actual return; in the no-enforcement condition, the selected responder can choose an actual return after matching, allowing declared and actual returns to differ. This design allows us to observe not only the sender’s trust investment and the responder’s actual return, but also the declared return that precedes matching and the gap between declaration and performance. This controlled experimental design, by allowing us to isolate the causal effects of competition and contract enforcement while holding all other factors constant, provides a clean identification strategy that is unattainable in naturally occurring field data.

Our results show that competition is the primary driver of trust in declaration-based exchange. Responder-side competition significantly increases the amount sent by senders and raises realized trustworthiness. By contrast, contract enforcement alone does not significantly increase trust as measured by the sender’s amount sent. Its main effect emerges when it is combined with competition: the interaction between competition and contract enforcement is positive and significant, indicating that enforceable contracts amplify the trust-enhancing effect of competition. Mechanism evidence supports this interpretation. Competition induces responders to make higher declarations and makes declarations relevant for sender selection. However, when declarations are nonbinding, higher declared returns are only partially credible and may create a gap between expected and realized returns. Contract enforcement closes this gap by making competitive declarations binding. Dynamic evidence further suggests that senders update subsequent trust based on realized returns and the credibility of prior declarations.

This paper contributes to three strands of literature. First, it contributes to the literature on communication, declarations, and credibility in trust relationships. Nonbinding promises may communicate intentions and shape beliefs, but they are often only imperfectly credible (Brown et al., 2004; Charness & Dufwenberg, 2006; Y. Chen & Zhang, 2021), and uncertainty may create room for self-serving behavior by trustees (Fooken, 2023). We add to this literature by distinguishing declared returns from realized returns. This distinction allows us to show not only whether declarations affect trust, but also whether declarations are credible, whether they are fulfilled, and how institutional enforceability changes the relation between what responders say and what they do.

Second, this paper contributes to the experimental literature on competition and trust. Existing evidence shows that competition can promote trust and market efficiency when partner choice is supported by information, reputation, or feedback mechanisms (Bolton et al., 2008; Huck et al., 2012), but other studies suggest that competitive incentives may weaken reciprocity or change how trusting behavior is perceived (Keck & Karelaia, 2012; Bauernschuster et al., 2013). We add to this literature by isolating responder-side competition in a declaration-based environment. Our findings show that competition is not merely a background market condition; it is the main force that increases sender investment and realized trustworthiness. At the same time, its effect is strongest when declarations are enforceable, showing that competition and credibility are complementary rather than interchangeable.

Third, this paper contributes to the literature on institutions, contracts, and trust. Prior work emphasizes that market outcomes depend not only on competition itself, but also on the institutional rules and information structures that make reliable behavior observable and enforceable (Brown et al., 2004; Cabrales et al., 2011). Our results refine this view by showing that contract enforcement does not simply or independently create trust. Instead, its central role is to amplify the trust-enhancing effect of competition by eliminating the gap between declared and actual returns. This finding helps explain why formal enforcement may have limited effects in the absence of competitive pressure, while becoming powerful when it converts competitive claims into credible commitments.

The remainder of the paper proceeds as follows. Section 2 reviews the related literature and develops the hypotheses. Section 3 describes the experimental design, procedures, sample, and empirical specification. Section 4 presents the main treatment effects, the mechanism evidence on declarations and credibility, and the dynamic patterns of trust updating. Section 5 concludes with implications for trust, competition, and institutional enforcement in declaration-based exchange.

## 2. Related Literature

### 2.1. Declarations as Signal for Trust

The literature on promises and communication shows that nonbinding statements can affect behavior, but their credibility is limited and context-dependent. Promise keeping depends on reliance damage and the expected consequences of breach (Sengupta & Vanberg, 2023), while incomplete promises allow self-serving interpretation when contingencies are not specified (Mittlaender, 2024). Related evidence on belief-dependent preferences suggests that agents may use uncertainty and information acquisition strategically to avoid the psychological cost of disappointing others (Rimbaud & Soldà, 2024). These findings reinforce the idea that nonbinding declarations may convey information but remain imperfectly credible without contractual enforcement.

In trust-related contexts, Charness and Dufwenberg (2006) show that promises can increase cooperation by changing beliefs and creating psychological costs of disappointing others. However, promises do not automatically eliminate opportunism. Y. Chen and Zhang (2021) provide recent evidence that elicited promises in trust settings may have limited effects on trusting behavior and that a substantial share of trustees do not fully keep their promises. Closely related evidence comes from Casella et al. (2018), who show that competition raises nonbinding offers in a sender–receiver game but does not raise actual transfers or expected transfers. Competition therefore changes the interpretation of promises rather than making them literally credible.

This issue is closely related to studies of cheap talk, plans, and promises in trust games. Bracht and Feltovich (2009) show that cheap-talk declarations alone have little effect in the trust game, but they become more effective when combined with observation of the trustee’s previous behavior, suggesting that nonbinding statements gain credibility when supported by behavioral history. Schniter et al. (2013) show that cheap-talk promises can promote trust, but they are not fully reliable: in the first round, 16.6% of trustees were not trusted and 18.8% of trusted trustees broke their promises. They further show that cheap talk can also help repair trust, as untrusted trustees often used longer messages and near-equal-split promises to repair damaged trust.

Together, these studies imply that declarations may convey useful information, but they do not automatically translate into trustworthy behavior. Unlike studies of cheap talk or general promises, our experiment elicits a specific declared return amount and the corresponding actual return, allowing us to measure directly whether declarations are fulfilled. By combining this declared-actual distinction with responder-side competition and contract enforcement, we examine whether competition makes declarations more informative or encourages strategic overstatement, and whether enforcement closes the gap between stated and actual behavior.

### 2.2. Competition and Trust

The literature offers two competing views on the role of competition in market exchange. On the one hand, market participation and competitive exchange may strengthen cooperation by expanding repeated interactions, making reputation valuable, and rewarding reliable behavior (Henrich et al., 2010; Al-Ubaydli et al., 2013). On the other hand, market incentives may also weaken social preferences or moral constraints, activate more instrumental motives, crowd out moral concerns, or change the perceived meaning of prosocial behavior (Gneezy & Rustichini, 2000; Ferrin & Dirks, 2003; Reeson & Tisdell, 2010; Sandel, 2012; Falk & Szech, 2013; Xin, 2019). Cabrales et al. (2011) and Brown et al. (2004) show that competition structure, information, bargaining power, and relational incentives can improve efficiency in exchange environments. Thus, whether competition promotes prosocial behavior and improves market efficiency, or instead erodes moral values, depends on the institutional environment in which competition takes place.

In experimental literature on trust and competition, the first set of studies suggests that competition can discipline opportunism when trustors can identify and select more reliable partners. Bolton et al. (2008) find that competition improves trust and trustworthiness in an experimental online-trading environment, especially when feedback mechanisms help buyers distinguish between sellers. Huck et al. (2012) show that competition among trustees can largely eliminate trust problems when even minimal information about past behavior is available. These studies support the view that competition can foster trust when it rewards reliable performance.

The effect of competition also depends on the structure of competition. Keck and Karelaia (2012) use tournament incentives in a one-shot trust-game setting and separately introduce competition among trustors and among trustees. They find that tournament incentives can increase trust on the trustor side but reduce trustworthiness on the trustee side. Bauernschuster et al. (2013) provide a closely related three-person competitive trust-game design, but they examine trustor-side competition, whereas our study focuses on responder-side competition. They find that trustor-side competition reduces the trustee’s return amount, because trustor’s investment may be interpreted as strategically motivated rather than as genuinely kind. Hoffmann et al. (2021) use tournament incentives in a repeated investment game to study how competition affects trust. They find that competition can increase trust, but when endogenous reputation is possible, competition reduces information sharing among trustors and thereby weakens the reputation channel that supports trust.

The effect of competition on trust is therefore theoretically and empirically ambiguous depending on both the structure of competition and the information available for competition. It may improve partner selection and increase realized returns, but it may also encourage strategic overstatement when declarations are not enforceable. In our experiment, responder-side competition is introduced through competing return declarations, so competition can promote trust only if these declarations help senders identify responders who will actually behave trustworthily.

### 2.3. Contract Enforcement to Reduce Uncertainty in Trust

A related experimental literature studies how different forms of uncertainty affect trust and trustworthiness. Some studies introduce exogenous uncertainty into the trust game through random shocks, probabilistic outcomes, information asymmetry, or ambiguity. Uncertainty can allow both trustor and trustees to hide opportunistic behavior behind random events and creates room for self-serving behavior, thus making trust and reciprocity more difficult to sustain (Castillo & Leo, 2010; Vranceanu et al., 2012; Sofianos, 2022; Fooken, 2023). Güth et al. (2014) and Clots-Figueras et al. (2016) examine uncertainty or ambiguity in the multiplier and information structure of the trust game, while Eskinazi et al. (2023) study how ambiguity or uncertain endowments shape trusting decisions. These studies show that exogenous uncertainty changes both beliefs and incentives in trust-related exchange, although its behavioral effect depends on where the uncertainty enters the game.

The type of uncertainty most relevant to the present paper is performance uncertainty: the possibility that a trustee’s stated intention may not be implemented in later behavior (Bracht & Feltovich, 2009; Schniter et al., 2013). Contract enforcement can reduce performance uncertainty generated by separation between declaration and actual behavior. Brown et al. (2004) show that relational contracts can sustain cooperation in repeated market exchange. However, relational enforcement relies on repeated interaction, observable past behavior, and the possibility of terminating future exchange. When exchange is based on ex ante declarations and realized behavior is observed only after the sender has already invested, informal enforcement may be too weak to make declarations fully credible. Contract enforcement therefore plays a distinct role: it reduces the responder’s ex post discretion by making the declared return binding, thereby narrowing the gap between stated intentions and actual behavior. Porter et al. (2025) provide recent experimental evidence that outside enforcement can increase trust in exchange relationships, and that informal networks cannot fully compensate for the enforcement mechanisms absent under weak institutions. However, the relationship between formal enforcement and trust is not straightforward: enforcement may not automatically increase interpersonal trust and can sometimes substitute for, rather than foster, trust (Malhotra & Murnighan, 2002), may produce non-monotonic crowding effects on trust (Bohnet et al., 2001), or may signal distrust and reduce cooperation through the hidden costs of control (Falk & Kosfeld, 2006).

This literature motivates a different form of uncertainty in our setting: return uncertainty generated by the separation between declared and actual behavior. Unlike studies of cheap talk or general promises, we ask responders to declare a specific return amount and then observe whether this declared return is implemented in actual behavior. This creates a direct measure of uncertainty of trust investment process: senders must judge whether declared returns predict later reciprocity. By combining this declared-actual distinction with responder-side competition, our design examines whether competition makes declarations more informative or instead encourages strategic overstatement. This declared-actual distinction also provides the basis for examining how institutional enforcement changes the credibility of declarations.

## 3. Materials and Methods

### 3.1. Experimental Design

This section describes the experimental design with the treatment setting, basic decision-making tasks, and payoff structure. The statistical analyses were conducted using Stata 18.0.

The experiment builds on the investment game of Berg et al. (1995) by introducing competition between responders. In each group, one sender interacts with two responders. The sender is endowed with CNY 10 and chooses how much to send. As in the standard trust game, the amount sent is tripled. After observing the amount sent, the responders independently determine return amount, with no initial endowment. The sender is then matched with a responder. Depending on the experimental treatment, this could be a sender’s choice or a random matching by the program, and it will be detailed in the following treatment description. The payoff is calculated as follows: the sender receives 10 − X + Y, where Y is the amount returned by the responder matched with the sender in the group. The successfully matched responder receives 3X – Y, and the unsuccessfully matched responder receives 0. This experiment uses a neutral framework, describing the sender and responder as participant A and participant B, respectively. The experiment will be repeated five times, with the payment from one round randomly selected as the final reward.

The key treatment variation concerns two dimensions: competition between responders and contract enforcement. These two dimensions generate a 2 × 2 design with four treatments. The first dimension is competition. To identify the effect of responder’s competition, we compare a competitive selection mechanism with a random-matching process. In the treatment without responder’s competition, the computer randomly assigns one of the two responders to the sender and calculates payoffs. The sender decides on the sent amount, and then, the responder decides on the declared return amount, respectively. In the competition treatments, the two responders compete for the sender’s investment opportunity. The sender observes the declared return amounts reported by both responder and chooses which responder to match with, and only the chosen responder has the opportunity to earn a positive payoff in that round. This competition dimension is implemented as a within-subject design: the same participant experiences both the competitive and the randomly matched environment.

The second dimension involves contractual enforcement. In the treatments of contract enforcement, the return contract is executed automatically. Once the reported return is submitted by the responder, the contract is automatically implemented. In the treatments without automatic contract execution, the reported return was only a declaration, with another actual return opportunity after the matching process. Specifically, the responder’s return decision-making process consisted of two phases. First, after observing the sender’s investment, each responder reports a declared return amount, and the sender matches with a responder. Second, the matched responder then submits the actual return amount that determines payoffs. This design allows declarations to differ from actual returns in the non-contract treatments, leaving room for responders to overstate their intended returns. For this dimension, the experiment employed a between-subjects design.

Combining these two dimensions yields four treatments. In Baseline, there is no competition among responders and no contract automatic execution. In Contract Only, the return contract is executed automatically, where sender matches with responder randomly. In Competition Only, responders compete for matching while declared return offer would not be executed automatically. In Competition and Contract, there is competition among respondents, and all return contracts are executed automatically. This 2 × 2 design allows us to identify the effects of competition, the effects of contractual enforceability, and their interaction. Table 1 summarizes the treatment settings. For brevity, we refer to these four treatments as B (Baseline), Cp (Competition Only), Ct (Contract Only), and CpCt (Competition and Contract) throughout the remainder of the paper.

### 3.2. Hypotheses

We develop several testable hypotheses around the main treatment effects and two mechanisms. The main treatment effects concern whether responder-side competition and contract enforcement increase trust and realized trustworthiness in a trust game with prior responder declarations. The mechanism hypotheses then examine how competition works through declared returns and sender selection, and how contract enforcement changes the credibility of these declarations.

First, responder-side competition changes the sender’s choice environment. In the random-matching treatments, the sender cannot choose between the two potential responders. In the competition treatments, by contrast, the sender observes both declared returns and can select the responder who appears more attractive. Prior market and trust-game experiments suggest that partner choice and competition can discipline opportunism when senders can identify and select more reliable partners (Bolton et al., 2008; Huck et al., 2012), although competition can have different behavioral consequences depending on its structure (Keck & Karelaia, 2012; Bauernschuster et al., 2013). In our setting, responders have a direct incentive to behave more trustworthily in order to attract the sender’s investment. We therefore expect competition to increase both the sender’s amount sent and responders’ realized returns.

**Hypothesis** **1.** *Responder competition increases trust and realized trustworthiness. Relative to random matching, competition should increase the amount sent because responders have stronger incentives to offer attractive returns and senders can select between alternative responders.*

Second, the effect of competition should depend on whether declared returns are enforceable. Without contract enforcement, a declaration is nonbinding: it may signal intended reciprocity, but the selected responder can later choose a different actual return. With contract enforcement, the declaration is automatically implemented and therefore becomes binding. Prior studies show that nonbinding communication can support cooperation, but its effect is limited when statements are not backed by observed behavior or enforcement (Charness & Dufwenberg, 2006; Bracht & Feltovich, 2009; Schniter et al., 2013; Y. Chen & Zhang, 2021). Related evidence also suggests that uncertainty creates room for self-serving behavior, while enforcement mechanisms can sustain cooperation when voluntary fulfillment is uncertain (Brown et al., 2004; Fooken, 2023; Porter et al., 2025). In our setting, contract enforcement makes declared returns binding and reduces fulfillment uncertainty. Competition should therefore have its strongest effect on trust and realized trustworthiness when declared returns are enforceable.

**Hypothesis** **2.** *Contract enforcement strengthens the effect of competition on trust and realized trustworthiness. Enforcement reduces fulfillment uncertainty by converting the declared return into an implemented return; therefore, the competition-and-contract treatment should produce the highest investment and return levels.*

The remaining hypotheses specify the mechanisms behind these treatment effects. The first mechanism concerns competition and declaration signaling. In competitive settings, declarations may be used strategically to attract selection, and competition can increase nonbinding offers without necessarily making them fully credible (Casella et al., 2018). In our competition treatments, declared returns are observable before responder selection and can therefore function as competitive signals. We expect competition to increase declared returns, and higher declarations to increase the probability of being selected.

**Hypothesis** **3a.** *Competition increases declared returns. Responders declare higher return amounts when they compete for the sender’s investment opportunity.*

**Hypothesis** **3b.** *Higher declarations increase the probability of selection. When sender choice is possible, responders who declare higher returns should be more likely to be selected.*

The second mechanism concerns contract enforcement and declaration credibility. Evidence on cheap talk, promises, and uncertainty in trust games suggests that nonbinding statements may convey information but remain imperfectly credible when later behavior can deviate from stated intentions (Charness & Dufwenberg, 2006; Bracht & Feltovich, 2009; Schniter et al., 2013; Y. Chen & Zhang, 2021; Fooken, 2023). In our setting, declared returns should contain information about intended reciprocity, but nonbinding declarations may be only partially credible. Contract enforcement should close this declared-actual gap by making the declared return automatically implemented.

**Hypothesis** **4a.** *Declared returns are informative but partially credible signals without contract enforcement. Declared returns should be positively associated with actual returns, but actual returns may be systematically lower than declared returns without contract enforcement.*

Finally, the repeated structure of the experiment allows us to examine whether senders learn from realized declaration credibility. In repeated interactions, senders may update subsequent trust according to positive and negative feedback from previous rounds. A higher realized return provides positive feedback about the responder’s trustworthiness, whereas a larger gap between declared and actual returns provides negative feedback about declaration credibility. When contract enforcement is present, this negative effect should disappear because declared returns are automatically implemented as actual returns. Thus, declaration matters not only for selection at the matching stage, but also for dynamic updating after realized outcomes are observed.

**Hypothesis** **4b.** *Sender updates trust from realized declaration credibility. Higher actual returns in the previous round increase the next-round amount sent, whereas a larger gap between declared and actual returns should reduce subsequent sending.*

Together, these hypotheses map the experimental design onto the empirical analysis. Hypotheses 1 and 2 concern the main effects of competition, contract enforcement, and their interaction. Hypotheses 3a and 3b test the competition mechanism by examining whether competition increases declared returns and whether higher declarations increase the probability of selection. Hypotheses 4a and 4b test the contract enforcement mechanism by examining declaration fulfillment and subsequent sender learning.

### 3.3. Procedures, Sample, and Empirical Specification

The experiment was programmed in oTree 5.0 (D. L. Chen et al., 2016) and conducted in the Behavioral and Policy Laboratory of Zhejiang Gongshang University between February and September 2023. Twelve sessions were completed in total. Two sessions had 18 participants, while the remaining sessions had 21 participants, yielding 246 recruited subjects overall. Each session lasted approximately 20 min, and the average payment was about CNY 23 (approximately USD 3.26), based on the average exchange rate of USD 1 = CNY 7.0467 for the year 2023 as reported by the National Bureau of Statistics of China. The study followed standard ethical protocols and was approved by the Institutional Review Board (IRB) prior to data collection. All subjects signed the consent form and agreed to participate in the experiment. Participants first read the experimental instructions under the guidance of the experimenter and were clearly informed that their decision-making would directly affect their final reward. After the formal experiment and questionnaire, participants received payments based on their experimental earnings.

The design included repeated observations across rounds and stages. In the empirical analysis, standard errors are clustered at the group level, and alternative specifications add round controls, stage controls, and individual characteristics including age, gender, general trust, risk preference, self-reported competition preference, and ambiguity attitude.

We focus on five outcome variables. Amount Sent measures trust. Actual Amount Returned and Actual Proportion Returned measure realized trustworthiness. Declared Amount Returned and Declared Proportion Returned capture the mechanism by which responders attempt to attract the sender’s investment. Because declared and actual proportions are only defined for observations with positive investment, the proportion regressions are estimated on a smaller sample than the amount regressions.

## 4. Results

In this section, we report the results of our experiments. We begin by presenting the basic descriptive statistics. We then conduct a static analysis to identify the main treatment effects on trust and realized trustworthiness, followed by a dynamic analysis of trends across rounds. Next, we examine the underlying mechanisms through which competition and contract enforcement operate, first analyzing the role of declarations in competitive selection, and then assessing the credibility of declarations and the dynamic learning patterns they generate. We also explore whether the treatment effects vary with individual characteristics. Finally, we synthesize the evidence by systematically comparing our hypotheses with the empirical findings.

### 4.1. Descriptive Statistics

The nonparametric tests follow the experimental design. The competition dimension is a within-subject comparison and is tested using Wilcoxon signed-rank tests, comparing B with Cp and Ct with CpCt. The contract dimension is a between-subject comparison and is tested using Wilcoxon rank-sum tests, comparing B with Ct and Cp with CpCt. The outcome variables are aggregated to group-level treatment means before testing. Table 2 reports the descriptive statistics by treatment.

### 4.2. Main Treatment Effects

**Figure 1:**
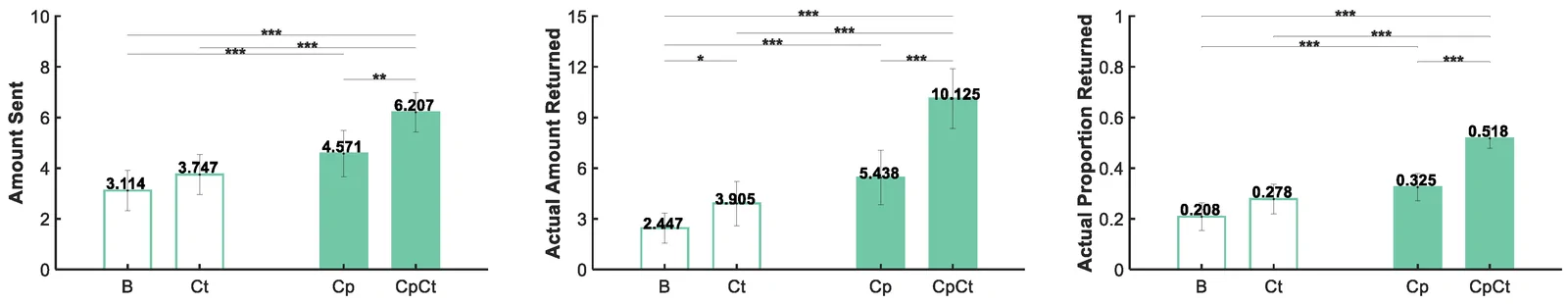
Main outcomes by treatment. Note: Error bars indicate 95% confidence intervals. Stars indicate significance levels from nonparametric tests. *, **, and *** denote significance at the 0.1, 0.05, and 0.01 levels, respectively. The same notation is used in the following bar charts.

Figure 1 shows a clear treatment effect for the three realized outcomes. For Amount Sent, the mean rises from 3.114 in the baseline treatment B to 4.571 in the competition treatment without contract enforcement (Cp), from 3.747 in the contract-only treatment (Ct) to 6.207 in the competition-and-contract treatment (CpCt). The within-subject Wilcoxon signed-rank tests confirm that competition significantly increases investment in both institutional environments: B versus Cp is significant (*p* = 0.0029), and Ct versus CpCt is also strongly significant (*p* < 0.001). On the contract dimension, investment does not differ significantly between B and Ct (*p* = 0.2279), but it is significantly higher in CpCt than in Cp (*p* = 0.0106) by Wilcoxon rank-sum test. The Wilcoxon rank-sum test also shows that investment in CpCt is significantly larger than that in baseline treatment B, which indicates the combined effect of competition and contract enforcement. Thus, competition robustly increases trust, while the positive effect of contract enforcement on sending behavior appears mainly when responders compete.

The same pattern is even more pronounced for Actual Amount Returned, which stands for the trustworthiness of the responder. Mean actual returns increase from 2.447 in B to 5.438 in Cp (*p* < 0.001), from 3.905 in Ct to 10.125 in CpCt (*p* < 0.001). On the contract dimension, the difference between B and Ct is marginal (*p* = 0.0697), whereas the gap between Cp and CpCt is large and highly significant (*p* < 0.001). The combined-treatment effect relative to the baseline is also highly significant between B and CpCt (*p* < 0.001). This pattern suggests that contract enforcement slightly increases realized trustworthiness under random matching, but it sharply strengthens the positive effect of competition.

Actual Proportion Returned tells the same story in relative terms of trustworthiness. Return proportion is defined conditional on a positive investment by the sender and calculated as the amount returned divided by three times the amount sent. When the sender invests zero, the responder can only return zero, so no meaningful return proportion can be computed, and such observations are excluded from the proportion-based analysis. Competition significantly increases the return proportion in both the non-contract environment from 0.208 in B to 0.325 in Cp (*p* < 0.001) and the contract environment from 0.278 in Ct to 0.518 in CpCt (*p* < 0.001). Contract enforcement alone does not significantly affect the return proportion under random matching (B versus Ct, *p* = 0.1172), but it significantly increases the return proportion when responders compete (Cp versus CpCt, *p* < 0.001). However, the combination effect is still highly significant (B versus CpCt, *p* < 0.001). Taken together, these figures indicate that competition raises both trust and trustworthiness, while contract enforcement amplifies the effect of competition.

To examine the effect of competition and contract enforcement on sender’s and responders’ behavior, we estimate random-effects regressions of the sender’s amount sent, responders’ actual amount returned and the actual return proportion. The key explanatory variables are indicators for Competition (Cp) and Contract (Ct), as well as their interaction. The specifications additionally control for round, stage, general trust, risk preference, competition preference, ambiguity preference, age, and gender.

The regression results are consistent with the descriptive evidence. Table 3 reports the treatment effects for the three main outcomes: Amount Sent, Actual Amount Returned and Actual Proportion Returned. In columns (1) and (2), the coefficient on Competition is positive and highly significant in the amount-sent regression (1.457, *p* < 0.01), indicating that competition between responders increases sender investment. Intuitively, competition improves the attractiveness of the environment from the sender’s perspective: when responders have to compete for the investment opportunity, they have stronger incentives to offer better terms, and senders respond by investing more.

The direct coefficient on Contract is positive but statistically insignificant for the Amount Sent, whereas the Competition × Contract interaction is positive and significant. This pattern suggests that contract enforcement alone does not generate the largest increase in trust directly; rather, competition is more effective at increasing sender investment when responders declared returns are binding. This finding suggests that competition can promote trust even in the absence of enforceable contracts, but the effect is amplified when market rules ensure that declared returns are credible.

The regressions for Actual Amount Returned reveal a broadly similar pattern for realized trustworthiness. In columns (3) and (4), the coefficient on Competition is again positive and highly significant, with an estimated effect of around 2.776 (*p* < 0.01). This indicates that responders return substantially more in competitive environments than in non-competitive ones. Thus, competition does not merely alter senders’ trust investment; it also affects actual behavior on the responder side. The coefficient on Contract is also positive and statistically significant in the return-amount regressions, implying that contract enforcement itself raises actual returns. This result indicates that once declared returns become binding, responders are not able to resubmit their returns downward after being selected. Most importantly, the interaction between Competition and Contract is positive and highly significant. This suggests that the strongest increase in actual return amounts occurs when competitive pressure is combined with contract enforcement. Competition encourages responders to offer better terms, while contract enforcement ensures that these favorable terms are actually implemented.

The return-proportion regressions in columns (5) and (6) reinforce the same conclusion. Competition significantly increases the actual proportion returned, and Contract also has a positive effect, although the magnitude is more modest. Once again, the interaction term is strongly positive and precisely estimated. The consistency between the amount and proportion specifications is important because it shows that the treatment effects on actual return amount are not driven solely by larger sender transfers generating mechanically larger returns in levels. Instead, the treatment effect operates not only on the actual amount returned, but also on the return proportion. Although the proportion measure is available only for the subsample with positive investment, its significance remains informative. It suggests that conditional on the sender’s positive transfer, responders return a larger share of the received amount under the treatment factor, implying a stronger degree of reciprocity even after controlling for the scale of investment.

### 4.3. Dynamic Patterns of Realized Outcomes Across Treatments

**Figure 2:**
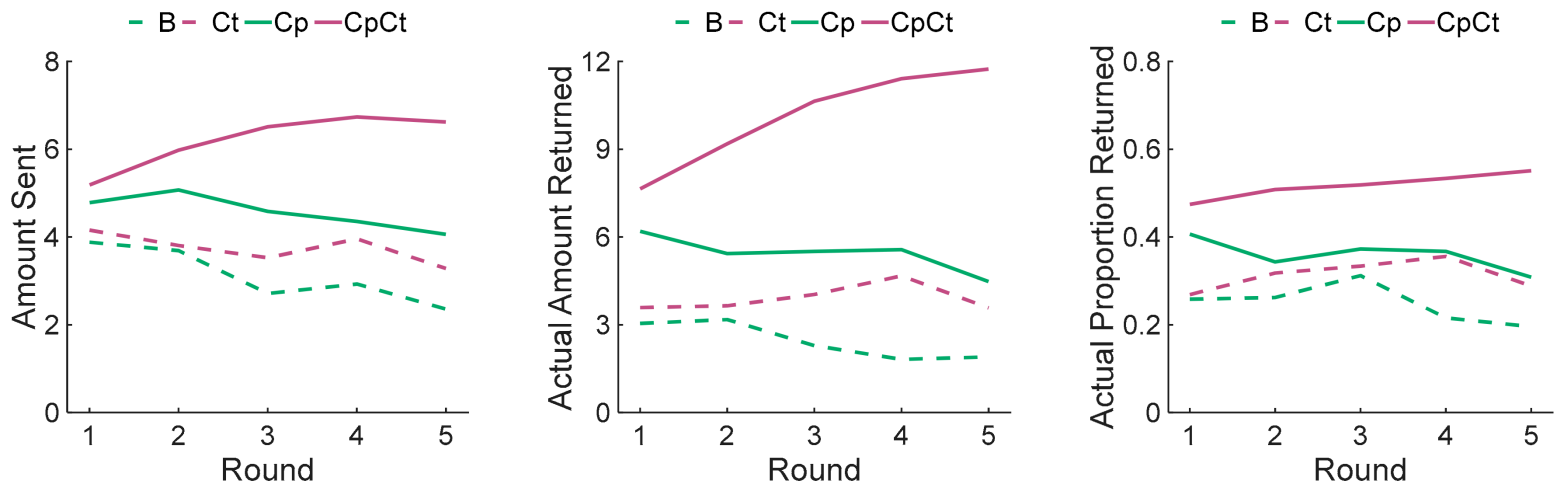
Dynamic patterns of trust and realized trustworthiness.

Figure 2 shows the dynamic trends. For Amount Sent, the baseline treatment exhibits a significant downward trend over rounds (ρ = −0.198, *p* = 0.0041), whereas the competition-and-contract treatment (CpCt) displays a significant upward trend (ρ = 0.158, *p* = 0.0257). The competition-only treatment and the contract-only treatment both show mild negative trends, but neither reaches conventional significance (Cp: ρ = −0.096, *p* = 0.1668; Ct: ρ = −0.098, *p* = 0.1660). This pattern suggests that trust erodes over repeated interaction in the baseline condition, whereas competition combined with contract enforcement strengthens trusting behavior over time.

For Actual Amount Returned, the strongest positive dynamic pattern again appears in CpCt. The return amount increases significantly over rounds in the competition-and-contract treatment (ρ = 0.219, *p* = 0.0018), while it decreases significantly in the competition-only treatment (Cp: ρ = −0.166, *p* = 0.0159). In the baseline treatment and the contract-only treatment, the correlations are negative but statistically insignificant (B: ρ = −0.113, *p* = 0.1013; Ct: ρ = −0.071, *p* = 0.3150). Thus, competition without contract enforcement does not induce progressively stronger trustworthiness. Instead, return amount increases over time only when competition is accompanied by contract enforcement.

The results for Actual Proportion Returned reinforce the same conclusion. Only CpCt shows a significant positive trend (ρ = 0.223, *p* = 0.0017), whereas the other three treatments do not display significant round-by-round changes (B: ρ = −0.054, *p* = 0.5405; Cp: ρ = −0.097, *p* = 0.2103; Ct: ρ = 0.060, *p* = 0.4650). Taken together, the dynamic tests indicate that the most stable and cumulative improvement in realized trust and trustworthiness occurs in the treatment that combines competitive selection with contract enforcement.

**Result 1: Competition can promote trust, and the effect is amplified when the declared contract is enforceable.**

**Result 2: Both competition among responders and contract enforcement increases realized trustworthiness, and contract strengthens the positive effect of competition.**

**Result 3: In the dynamic analysis, only the competition-and-contract treatment produces sustained upward trends in both the amount sent by senders and the amount returned by responders.**

### 4.4. Mechanism: Competition and Declaration

We first examine the competition-based mechanism. If competition promotes trust through declarations, it should increase responders’ declared returns and make those declarations relevant for sender selection. We therefore analyze whether competition raises declared returns and whether responders with higher declarations are more likely to be selected.

#### 4.4.1. Competition Increase Trust Through Declaration

**Figure 3:**
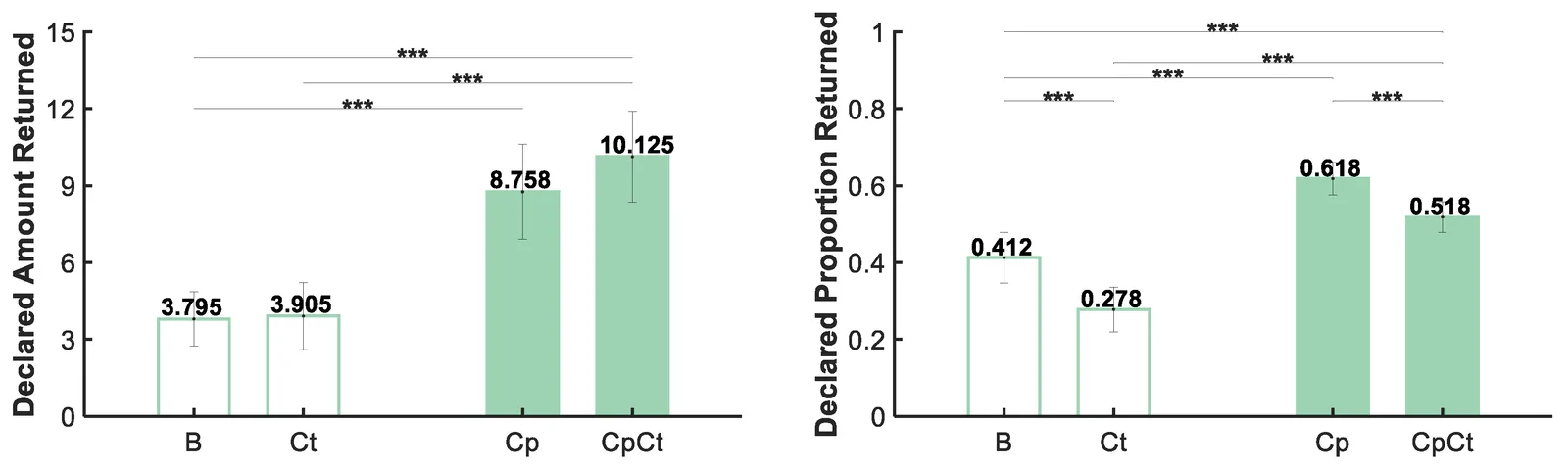
Declared return outcomes by treatment. Note: Error bars indicate 95% confidence intervals. Stars indicate significance levels from nonparametric tests. *** denote significance at the 0.01 levels.

We next examine the mechanisms through which the two institutions affect trust behavior. We first analyze how competition affects declared returns. Figure 3 shows that competition has a strong effect on declared outcomes. For Declared Amount Returned, the mean rises from 3.795 in B to 8.758 in Cp, remains low at 3.905 in Ct, and reaches 10.125 in CpCt. The signed-rank tests confirm that competition significantly increases declared return amounts both without contract enforcement (B versus Cp, *p* < 0.001) and with contract enforcement (Ct versus CpCt, *p* < 0.001). By contrast, contract enforcement does not significantly change declared return amounts under either matching regime (B versus Ct, *p* = 0.7869; Cp versus CpCt, *p* = 0.2355). The combined-treatment effect relative to the baseline is also highly significant between B and CpCt (*p* < 0.001). This pattern indicates that competition is the main driver of higher stated return amounts.

Declared Proportion Returned shows a similar but more nuanced pattern. Competition significantly increases declared proportions under both contract environments (B versus Cp, *p* < 0.001; Ct versus CpCt, *p* < 0.001). However, contract enforcement significantly lowers the declared proportion both under random matching (B versus Ct, *p* = 0.0038) and under competition (Cp versus CpCt, *p* = 0.0010). In other words, nonbinding environments encourage responders to promise a larger share of the tripled investment, especially when competing for selection, whereas binding implementation restrains such overstatement.

Table 4 reports the treatment effects for the declared outcomes: Declared Amount Returned and Declared Proportion Returned. Competition increases declared return amounts very strongly (4.962, *p* < 0.01), indicating that responders use declared returns as signals to attract the sender’s investment. By contrast, the coefficient on Contract (Ct) is small and statistically insignificant, suggesting that contract enforcement by itself does not induce responders to announce higher or lower return amounts. The interaction term between Competition and Contract is also positive but insignificant, implying that competition is the primary force driving upward declarations, while contract enforcement does not further amplify declared amounts once competition is already present. In other words, responders appear to use generous declarations as a competitive signal regardless of whether those declarations are binding.

The proportion regressions in columns (3) and (4) show a similar pattern. Competition again has a strong and highly significant positive effect (0.193, *p* < 0.01), indicating that responders under competition promise a markedly larger share of the tripled amount sent. Thus, whether declarations are measured in levels or proportions, competition intensifies upward signaling. However, unlike in the return amount regressions, contract now has a negative and significant effect (−1.250, *p* < 0.01). This implies that when declarations are binding, responders announce lower return proportions than in the baseline. One interpretation is that contract enforcement restrains overpromising: once declared returns are implemented automatically, responders are more likely to reveal their true intended level of reciprocity rather than strategically inflating their promises in order to secure the sender’s choice.

The fact that this effect is statistically significant for declared return proportions but not for declared return amounts may reflect sample selection. Declared return proportions are defined only for observations with positive investment, so these regressions are estimated on a selected subsample in which the sender has already chosen to send a positive amount. Within this subsample, responders still have room to lower the proportion they declare once promises become binding, and the disciplining effect of contract enforcement therefore becomes more visible. By contrast, the declared-amount regressions include all observations, including those in which the sender sends zero and the declared return is mechanically zero. Because these zero-send, zero-return observations are retained in the sample, the overall contract effect on declared amounts is diluted, which may explain why it is not statistically significant in the amount regressions.

#### 4.4.2. Dynamic Patterns of Declared Outcomes Across Treatments

**Figure 4:**
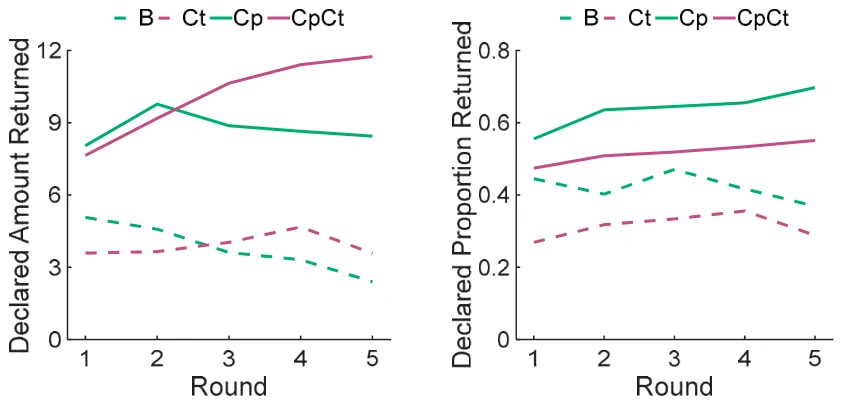
Dynamic patterns of declared return outcomes.

The dynamic patterns for declared outcomes highlight an important difference between signaling declaration and actual trustworthiness. Figure 4 shows the dynamic trends. For Declared Amount Returned, the baseline treatment shows a significant downward trend across rounds (ρ = −0.233, *p* = 0.0007), suggesting that responders gradually reduce their stated return amounts when neither competition nor contract enforcement is present. By contrast, CpCt still shows a significant upward trend (ρ = 0.219, *p* = 0.0018), whereas Cp and Ct remain essentially flat and statistically insignificant (Cp: ρ = −0.046, *p* = 0.5054; Ct: ρ = −0.071, *p* = 0.3150). This means that the most credible upward evolution in declarations again occurs only when competition is paired with enforceability.

For Declared Proportion Returned, the most notable result is that both competitive treatments show upward movement, but the pattern is stronger and more interpretable when contract enforcement is present. The declared proportion rises significantly in CpCt (ρ = 0.223, *p* = 0.0017) and also in Cp (ρ = 0.269, *p* = 0.0005). In contrast, neither B nor Ct shows a significant trend (B: ρ = −0.098, *p* = 0.2651; Ct: ρ = 0.060, *p* = 0.4650). The results imply that competition tends to intensify upward declarations, but only the competition-and-contract treatment turns those increasingly favorable declarations into sustained improvements in actual trustworthiness.

**Result 4a: The increase in the declared return amount is realized through competition among the responders.**

#### 4.4.3. Sender’s Selection on Higher Declared Return

The preceding results have shown that competition raises declared returns. We next examine whether these declarations are effective signals: are responders who declare higher return amounts more likely to be selected by senders? Table 5 examines the selection channel. The dependent variable is whether a responder is chosen, and the key explanatory variable is Declared Higher, an indicator equal to one when the responder declared a higher return than the other responder in the pair group. In the pooled specification, the coefficient on Declared Higher is positive and highly significant (0.329, *p* < 0.01), indicating that responders with higher declarations are more likely to be selected. The treatment-specific columns show that this effect is driven by the competition treatments. In the random-matching treatments, the coefficient is small and insignificant in B and Ct, as expected because the chosen responder is not determined by sender choice. In Cp, the coefficient is 0.392 and significant; in CpCt, it rises to 0.816 and is again highly significant. The interaction specification in column (6) is consistent with this pattern: the baseline effect of Declared Higher is insignificant, while its interactions with Cp and with the competition-contract condition are positive and significant. Thus, higher declarations matter for selection primarily when senders can choose between responders, and the selection effect is strongest when declarations are contractually credible.

**Result 4b: Higher declarations help responders compete for selection.** Responders use declared returns as competitive signals, and higher declarations increase the probability of being selected when sender choice is possible.

### 4.5. Mechanism: Declaration and Contract Enforcement

Since competition increases declared returns and senders tend to select responders who declare higher returns, the next question is whether these declarations translate into actual return behavior. Examining the relationship between declared and actual returns allows us to assess whether declarations are fulfilled and how contract enforcement changes this relationship. We further examine dynamic updating by testing whether the previous round’s declared and realized return affects the sender’s subsequent amount sent, and whether a larger declared-actual return gap reduces next-round investment. Together, these analyses clarify the role of contract enforcement by showing how it changes the relationship between declared and actual returns and how senders dynamically respond to realized returns and declared-actual gaps.

#### 4.5.1. Relationship Between Declared Return and Actual Return

This subsection examines the relationship between declared returns and actual returns and assesses whether the credibility of declarations differs between non-contract and contract-enforcement treatments. Table 6 examines whether declared returns are fulfilled in actual return behavior. Columns (1)–(3) use Actual Amount Returned as the dependent variable and include Declared Amount Returned and its squared term as the key explanatory variables. Columns (4)–(6) use Actual Proportion Returned as the dependent variable and include Declared Proportion Returned and its squared term. The treatment-specific specifications focus on B and Cp, where declarations are not automatically implemented. In columns (1)–(3), declared return amounts are positively and significantly associated with actual return amounts in the pooled, B, and Cp specifications, while the squared term is not statistically significant. This shows that higher declarations generally predict higher actual returns. The proportion regressions in columns (4)–(6) shows that declared return proportions have positive and significant linear effects, but the squared term is negative and significant in the pooled model and in Cp. This concave pattern indicates that actual return proportions increase with declared proportions at low and moderate levels, but the marginal fulfillment of very high declarations declines, especially under competition without enforcement.

**Figure 5:**
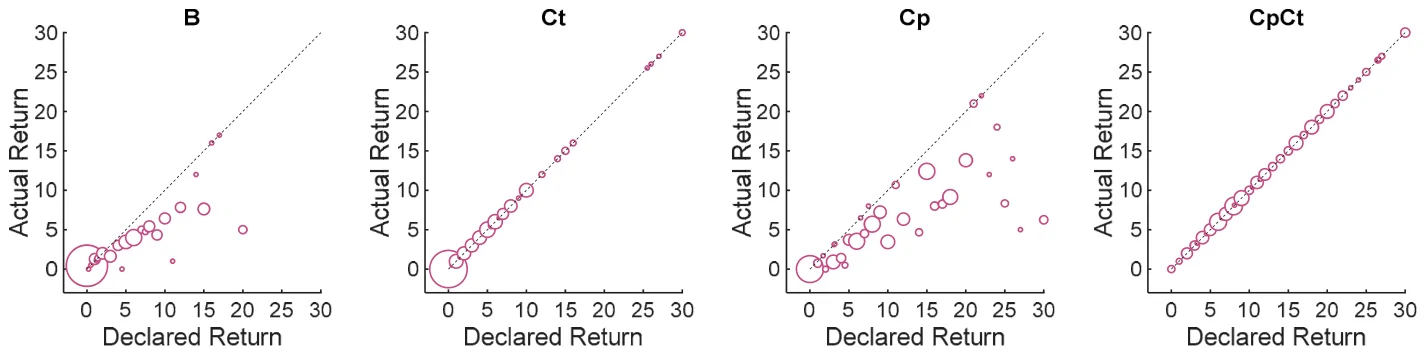
Declared and actual returns by treatment. Note: The size of each circle represents the number of observations at the corresponding combination of declared and actual returns. The 45-degree dashed line indicates equality between declared and actual returns.

This is also visible in Figure 5. Both the regression results and the graphical patterns indicate that actual returns are generally lower than declared returns in the non-contract treatments. In the contract-enforcement treatments Ct and CpCt, declared returns are mechanically implemented as actual returns because the contract makes the declared return binding. Contract enforcement therefore eliminates the gap between promised and realized returns. In these treatments, the downward curvature observed in the non-contract treatments no longer arises: high declarations are no longer merely cheap signals that may be partially credible, but enforceable commitments. By contrast, in B and especially in Cp, declarations remain nonbinding, so higher declared returns can still be followed by lower realized returns, generating the credibility gap.

**Result 5a: Nonbinding high declarations are only partially credible.** Declared returns are positively associated with actual returns, but the declarations become fully credible only under contract enforcement.

#### 4.5.2. Amount Sent and Lagged Return Amount

Table 7 focuses on dynamic part of the mechanism by investigating whether the sender’s next-round Amount Sent responds to previous-round return experience. Column (1) includes the lagged declared return amount received by the sender as an explanatory variable. The coefficient of is positive and significant (0.109, *p* < 0.01), suggesting that a higher promised return in the previous round is associated with higher subsequent investment. Column (2) replaces this variable with the lagged actual return amount received. The coefficient is larger and highly significant (0.237, *p* < 0.01), indicating that senders respond strongly to realized reciprocity. Column (3) includes the lagged declared return amount and the lagged actual return amount simultaneously. Once the actual return is controlled for, the lagged declared return becomes small and statistically insignificant (−0.026), whereas the lagged actual return remains positive and highly significant (0.250, *p* < 0.01). This result indicates that the association between declarations and future trust operates mainly through whether the declaration is actually fulfilled.

Column (4) includes the lagged gap between declared and actual return amounts, defined as the declared return amount minus the actual return amount in the previous round. The coefficient is negative and significant (−0.149, *p* < 0.01), showing that a larger gap between what was declared and what was actually returned reduces the sender’s next-round investment. This result indicates that senders learn from realized credibility: fulfilled declarations support future trust, whereas overstatement depresses subsequent trust.

Columns (5) and (6) further examine whether senders respond to their own prior investment outcomes. Column (5) includes the lagged gap between the sender’s own amount sent and the actual return received, defined as the previous-round amount sent minus the actual return received. This variable captures the continuous size of the sender’s realized loss from the previous round. The coefficient is negative and highly significant (−0.237, *p* < 0.01), indicating that when the sender’s previous investment exceeds the return received by a larger amount, the sender reduces the next-round investment more strongly. Column (6) replaces this continuous gap with a loss indicator, which equals one if the sender’s previous amount sent is greater than the actual return received, and zero otherwise. The coefficient is also negative and highly significant (−2.108, *p* < 0.01), showing that experiencing a loss in the previous round substantially lowers the sender’s next-round amount sent. Together, these results suggest that senders not only respond to whether responders fulfill their declarations, but also update future trust based on whether their own prior investment produced a net loss.

These findings provide behavioral evidence consistent with learning and trust calibration from prior return experience. Although we do not directly measure senders’ expectations, the dynamic results show that senders adjust their next-round investment according to realized outcomes in the previous round. Higher actual returns reinforce subsequent trust, whereas larger declared-actual gaps and sender-side losses lead senders to reduce future investment, consistent with downward trust recalibration after unfavorable return experience.

Treatment-specific subsample regressions for the lagged return-gap and sender-loss measures are reported in Appendix B, and the results are consistent with the pooled regressions.

**Result 5b: Sender investment reflects behavioral learning from previous-round return experience. Senders adjust their next-round investment in response to realized outcomes: higher actual returns increase subsequent investment, whereas larger declared-actual gaps and sender-side losses lead senders to reduce future trust.**

### 4.6. Moderation Analysis

Table 8 reports exploratory moderation analyses for sender investment. The purpose is to examine whether the treatment effects on Amount Sent vary with individual characteristics. We focus on the two moderators that show significant additional interaction effects: gender and ambiguity attitude.

Column (1) interacts Gender with the full treatment structure, where Gender = 1 indicates male participants. The main effect of competition remains positive and significant, indicating that competition increases Amount Sent among female participants, the reference group. More importantly, the triple interaction Gender × Cp × Ct is positive and significant (2.710, *p* < 0.05). This suggests that the joint effect of competition and contract enforcement is stronger for male participants than for female participants.

Column (2) conducts the same analysis for ambiguity preference. This variable is measured using a binary choice between a risky box with a known composition and an ambiguous box with an unknown composition. Participants choosing the known-risk box are coded as 0, whereas those choosing the ambiguous box are coded as 1. Thus, Ambiguity = 1 indicates ambiguity-seeking participants. The coefficient on Cp remains positive and significant, and the triple interaction Ambiguity × Cp × Ct is positive and significant (2.614, *p* < 0.05). This indicates that the trust-enhancing effect of combining competition with contract enforcement is stronger among ambiguity-seeking participants than among participants who prefer the known-risk option.

These moderation results reinforce the main interpretation of the experiment. Competition and contract enforcement work together to increase trust, but the magnitude of this institutional synergy differs across individual characteristics. We also examined the remaining control variables as moderators of Amount Sent, as well as all control variables as moderators of Actual Amount Returned, but found no significant three-way interaction effects.

Taken together, the mechanism results show that competition and contract enforcement promote trust through declared returns. Responders use declarations to compete for selection, and senders respond to higher declarations when selection is possible. Declared returns are generally positively associated with actual returns, suggesting that declarations contain credible information about responders’ intended reciprocity, but the declarations become fully credible only under contract enforcement.

Sender behavior in later rounds confirms that realized returns, rather than declarations alone, sustain trust over time. Moreover, the declared-actual return gap is negatively associated with the next-round amount sent, indicating that declaration fulfillment is crucial for maintaining trust. Thus, competition creates a declaration-signaling channel that raises responders’ declared returns and realized return behavior, and this channel is stronger and more sustainable when declarations are credible. Contract enforcement closes the declared-actual return gap by making declared returns binding, thereby aligning competitive declarations with actual behavior and generating the strongest increase in trust when combined with competition.

The dynamic results further show that senders learn from previous-round return experience: sender-side losses reduce next-round investment. This pattern reflects behavioral learning and trust calibration: senders adjust their future investment according to prior return experience, increasing trust when realized reciprocity is credible and reducing trust when previous outcomes reveal unfulfilled promises or personal losses.

The moderation results further suggest that this institutional synergy is not uniform across individuals: the combined effect of competition and contract enforcement on sender investment is stronger among male and ambiguity-seeking participants, indicating meaningful heterogeneity in how participants respond to credible competitive environments.

### 4.7. Hypotheses and Empirical Evidence: A Synthesis

Before turning to the general discussion, we summarize the relation between our theoretical predictions and the empirical evidence. Table 9 reports, for each hypothesis developed in Section 3.2, the predicted outcome, the corresponding empirical finding, and whether the hypothesis is supported by the data. This synthesis serves two purposes: it provides a systematic overview of how our experimental results align with the theoretical framework, and it clarifies which patterns emerged as expected versus which required more nuanced interpretation.

As shown in Table 9, the experimental results confirm all six hypotheses. Competition robustly increases trust and realized trustworthiness (H1), and its effect is amplified by contract enforcement (H2). The mechanism analysis supports the competition-based signaling channel (H3a, H3b): competition raises declared returns, and higher declarations increase the probability of being selected when sender choice is possible. The credibility-based channel (H4a, H4b) is also supported: declarations are positively associated with actual returns but are only partially credible without enforcement, and senders update subsequent trust based on realized returns and the declared-actual gap.

Two patterns deserve particular attention. First, the support for H2 is asymmetrical: enforcement alone does not significantly increase trust (the main effect of Contract on Amount Sent is insignificant), but its interaction with Competition is positive and significant across all three outcome measures. This pattern confirms that enforcement functions primarily as an amplifier rather than an independent driver of trust—a distinction that we elaborate in the discussion. Second, the support for H4a reveals a concave relationship in the competition-without-enforcement treatment (Cp): declared proportions predict actual proportions at moderate levels, but the marginal credibility of very high declarations declines. This suggests that competitive pressure without enforcement encourages strategic overpromising, which is precisely the behavioral mechanism that contract enforcement resolves.

Overall, the evidence demonstrates that the two institutional mechanisms operate through distinct but complementary channels: competition generates attractive declarations and selection incentives, while enforcement ensures that these declarations translate into credible commitments. The combination of the two produces the strongest and most sustained trust outcomes.

## 5. Discussion and Conclusions

### 5.1. Institutional Synergy: Competition as the Engine, Enforcement as the Amplifier

With all six hypotheses confirmed (Table 9), we now turn to a broader interpretation of our findings. The static and dynamic results consistently demonstrate an asymmetric synergy between the two institutional pillars: responder-side competition operates as the primary engine driving market trust, whereas contract enforcement functions as an indispensable credibility amplifier.

As detailed in our baseline and single-mechanism treatments, contract enforcement alone (Ct) does not generate a statistically significant increase in trust investments relative to the baseline (B). This pattern is clearly reflected in the regression results: the main effect of Contract on Amount Sent is positive but insignificant (Table 3, columns 1–2), whereas the Competition × Contract interaction is positive and significant across all three outcome measures. In the absence of competitive pressure, potential responders lack market-driven incentives to signal high returns; consequently, enforceable contracts merely tie responders to a modest baseline level of reciprocal yield. Conversely, competition alone (Cp) successfully unleashes the primary engine of the market: responders intensely outbid one another by declaring significantly higher returns to secure selection, stimulating the sender’s initial investment. However, without contractual backing, these competitive claims degenerate into cheap-talk signals that are only partially credible, generating a declaration-fulfillment gap.

The maximum level of market trust and realized trustworthiness is unlocked exclusively when competition is paired with contract enforcement (CpCt). Enforcement provides the structural enclosure needed to secure competitive signaling, converting attractive promises into non-discretionary commitments.

### 5.2. Behavioral and Cognitive Mechanisms: Expectation Disconfirmation and Trust Calibration

Moving beyond the institutional main effects, our dynamic analysis clarifies the psychological channels through which market participants calibrate trust over time. By embedding our dynamic results (Table 7) in Expectation Confirmation Theory (ECT) (Oliver, 1980; Lankton & McKnight, 2012) and trust calibration frameworks (McKnight et al., 2002; Okamura & Yamada, 2020), we trace the psychological trajectory of trust erosion.

In declaration-based exchanges, responders’ prior announcements establish a cognitive baseline for the trustor’s expectations. Neuroeconomic evidence suggests that such subjective expectations activate reward-processing networks in the bilateral ventral striatum (Fareri et al., 2012; Fairley et al., 2019). When competition is introduced without enforcement (Cp), the rivalry for selection inflates responders’ declarations, elevating the sender’s expectation baseline. Ex post, however, the absence of enforcement allows responders to underdeliver on their promises. This discrepancy generates a negative expectation disconfirmation—psychological disappointment and a downward recalibration of trust. In neuroeconomic terms, the declaration-fulfillment gap triggers a negative reward prediction error, diminishing the subjective value of future interactions.

Our dynamic modeling (Table 7, Column 4) confirms this behavioral mechanism: the previous round’s return-gap exerts a significant negative coefficient on subsequent investments. Senders do not learn from declarations alone; they learn from realized credibility. When competitive claims are repeatedly falsified, trust collapses, driving senders toward zero investment—as observed in the declining extensive margin in the Cp treatment. Contract enforcement solves this decay by eliminating performance uncertainty, aligning expectations with outcomes.

Our experimental design provides a direct empirical setting for observing trust calibration—the process by which trustors adjust their trust to align with the realized trustworthiness of trustees (McKnight et al., 2002; Okamura & Yamada, 2020). In each round, senders observe two signals: the responder’s declared return (which establishes an expectation) and the actual return received (which reveals whether the expectation was met). The gap between these two signals—our return-gap measure—constitutes a direct behavioral proxy for expectation disconfirmation. As shown in Table 7, senders systematically update their subsequent trust based on this gap: a larger gap reduces next-round investment, indicating that trust is calibrated downward when prior expectations are violated. This calibration process is asymmetrical, however: senders respond strongly to realized returns but not to declarations alone (Table 7, Column 3), suggesting that trust calibration in declaration-based exchange is driven primarily by realized rather than stated credibility. Contract enforcement effectively removes the need for downward calibration by eliminating the gap, thereby stabilizing trust at a higher equilibrium. Thus, our findings offer direct behavioral evidence for trust calibration in a controlled market environment, a phenomenon that has largely been studied theoretically or in human–computer interaction contexts.

A related behavioral dimension concerns the trade-off between strategic freedom and institutional rigidity. Low-enforcement environments may feel more engaging, as participants retain autonomy to make voluntary choices. In our Baseline (B) and Competition Only (Cp) treatments, any return represents a genuine manifestation of intrinsic motivation or voluntary reciprocity. However, when this freedom is combined with competitive pressure, it invites strategic overpromising, crowding out intrinsic cooperation and generating subsequent disappointment. Enforcement imposes rigidity by restricting ex-post discretion, but this very constraint provides trustors with psychological safety—a structural protection against betrayal. By converting uncertain competitive signals into binding commitments, enforcement alleviates betrayal aversion and stabilizes exchange.

We acknowledge that these psychological and neural mechanisms were not directly measured in our experiment; our interpretations are inferential, grounded in observed behavioral patterns. Nevertheless, they provide a generative framework for future research combining experimental economics with process-tracing methods such as fMRI, pupillometry, or subjective expectation elicitation.

### 5.3. Practical Implications, Limitations, and Future Directions

These findings offer practical guidance for digital marketplaces, peer-to-peer lending platforms, and crowdfunding networks. Platforms often stimulate seller competition through flexible return policies, advertised quality, or repayment promises. Our results caution that promoting unconstrained competition without robust verification infrastructure triggers strategic overpromising, consumer disappointment, and platform-wide trust decay. To sustain platform health, operators should implement formal contract structures—such as automated escrow accounts, smart contracts, or mandatory performance bonds—that transform competitive slogans into binding obligations.

Several limitations point to fruitful directions for future research. First, our sample comprised 246 participants, all of whom were university students. Although student samples in standardized investment games have been shown to exhibit decision-making processes structurally similar to those of the general population, future field experiments could validate these mechanisms among market professionals. Second, our experiment was conducted in a single cultural context with relatively high baseline social trust. Cross-cultural replications would help establish whether the competition-enforcement synergy generalizes to societies with weaker social capital. Third, future extensions could introduce costly or imperfect enforcement, multilateral repeated matching, and endogenous reputation systems to examine how formal institutions and informal networks interact over longer horizons.

A further limitation concerns the statistical sensitivity of the study. To characterize the sensitivity of the realized sample, we conducted sensitivity power analyses using G*Power 3.1.9.7 with two-sided tests, an alpha level of 0.05, and 80% power. For the paired comparison between Baseline and Competition Only involving 42 matched groups, the Wilcoxon signed-rank test could detect a standardized paired effect of d = 0.454. For the independent comparisons between Baseline (N = 42) and Contract Only or Competition and Contract (N = 40), the Wilcoxon–Mann–Whitney test could detect an effect of d = 0.642. Relative to these sensitivity benchmarks, the observed Baseline–Competition Only effects were d = 0.518 for Amount Sent and d = 0.711 for Actual Return Amount, while the corresponding Baseline–Competition and Contract effects were d = 1.233 and d = 1.757. By contrast, the Baseline–Contract Only effects were smaller, with d = 0.252 for Amount Sent and d = 0.416 for Actual Return Amount. Overall, the sensitivity analysis indicates that the realized design had adequate sensitivity to detect effects in the range observed for the competition-only and combined-treatment contrasts, supporting the principal findings of the study. The smaller contract-only estimates should be interpreted more cautiously, as effects below the detectable range may be estimated with less precision rather than indicating the absence of an effect. Future research with larger samples and additional independent experimental sessions could further improve estimation precision and assess smaller institutional effects.

### 5.4. Conclusions

This study demonstrates that prior declarations can support trust in economic exchange, provided they are structured by appropriate institutional architecture. Our findings show that competition is the indispensable engine that generates high declared returns and stimulates sender investment. However, competition alone cannot sustain cooperation; without contractual enforcement, competitive pressures trigger strategic overpromising and psychological disconfirmation, eroding trust over time. Contract enforcement serves as the critical amplifier that removes performance uncertainty, eliminates the declaration-fulfillment gap, and anchors trustor expectations. Ultimately, the most robust and sustainable market relationships emerge not from either mechanism in isolation, but from their deliberate institutional integration.

## Figures and Tables

**Table 1:** Treatment settings.

	Contract: No	Contract: Yes
**Competition: No**	Baseline (B)	Contract Only (Ct)
**Competition: Yes**	Competition Only (Cp)	Competition and Contract (CpCt)

**Table 2:** Descriptive statistics by treatment.

Treatment	Statistic	Amount Sent	Actual Amount Returned	Declared Amount Returned	Actual Proportion Returned	Declared Proportion Returned
B	Mean	3.114	2.447	3.795	0.208	0.412
	SD	2.549	2.834	3.390	0.168	0.200
	*N*	42	42	42	38	38
Cp	Mean	4.571	5.438	8.758	0.325	0.618
	SD	2.921	5.188	5.963	0.175	0.134
	*N*	42	42	42	42	42
Ct	Mean	3.747	3.905	3.905	0.278	0.278
	SD	2.459	4.097	4.097	0.175	0.175
	*N*	40	40	40	37	37
CpCt	Mean	6.207	10.125	10.125	0.518	0.518
	SD	2.462	5.543	5.543	0.124	0.124
	*N*	40	40	40	40	40

**Table 3:** Main treatment effects on trust and realized trustworthiness.

	(1)	(2)	(3)	(4)	(5)	(6)
	Amount Sent	Amount Sent	Actual Amount Returned	Actual Amount Returned	Actual Proportion Returned	Actual Proportion Returned
Cp	1.457 ***	1.457 ***	2.765 ***	2.776 ***	0.101 ***	0.101 ***
	(0.409)	(0.410)	(0.643)	(0.645)	(0.032)	(0.032)
Ct	0.632	0.547	1.460 **	1.504 **	0.059 *	0.063 *
	(0.510)	(0.478)	(0.744)	(0.751)	(0.036)	(0.035)
Cp × Ct	1.003 *	1.003 *	3.455 ***	3.444 ***	0.121 ***	0.120 ***
	(0.575)	(0.577)	(0.990)	(0.994)	(0.037)	(0.037)
Constant	4.857 ***	5.464 *	3.266 ***	2.099	0.274 ***	0.321 **
	(0.676)	(2.944)	(1.055)	(3.638)	(0.036)	(0.155)
Stage and Round Controls	Yes	Yes	Yes	Yes	Yes	Yes
Individual Controls	No	Yes	No	Yes	No	Yes
Observations	820	820	1220	1220	992	992
Subjects	82	82	164	164	160	160

Notes: Clustered standard errors in group level in parentheses. Stage and round controls include round and stage. Individual controls include age, gender, general trust, risk preference, competition-report, and ambiguity. *, **, and *** denote significance at the 0.1, 0.05, and 0.01 levels, respectively. The following tables use the same notation, controls, and standard-error specification.

**Table 4:** Treatment effects on declared return outcomes.

	(1)	(2)	(3)	(4)
	Declared Amount Returned	Declared Amount Returned	Declared Proportion Returned	Declared Proportion Returned
Cp	4.962 ***	4.962 ***	0.194 ***	0.193 ***
	(0.866)	(0.868)	(0.032)	(0.032)
Ct	0.110	0.145	−0.130 ***	−0.125 ***
	(0.784)	(0.787)	(0.040)	(0.038)
Cp × Ct	1.257	1.257	0.023	0.023
	(1.149)	(1.151)	(0.037)	(0.036)
Constant	5.403 ***	0.839	0.410 ***	0.257 *
	(1.159)	(3.676)	(0.032)	(0.151)
Stage and Round Controls	Yes	Yes	Yes	Yes
Individual Controls	No	Yes	No	Yes
Observations	1640	1640	1290	1290
Subjects	164	164	164	164

Notes: Clustered standard errors in group level in parentheses. * and *** denote significance at the 0.1 and 0.01 levels, respectively.

**Table 5:** Responder selection based on higher declared returns.

	(1)	(2)	(3)	(4)	(5)	(6)
	Pooled	B	Cp	Ct	CpCt	Med
Declared_Higher	0.329 ***	−0.006	0.392 ***	0.053	0.816 ***	0.001
	(0.034)	(0.060)	(0.064)	(0.066)	(0.036)	(0.062)
Cp						−0.130 ***
						(0.030)
Ct						−0.021
						(0.028)
Cp × Ct						−0.200 ***
						(0.042)
Declared_Higher × Cp						0.358 ***
						(0.089)
Declared_Higher × Ct						0.044
						(0.089)
Declared_Higher × Cp × Ct						0.412 ***
						(0.111)
Constant	0.229	0.604	0.451	0.446	−0.373	0.518 ***
	(0.199)	(0.428)	(0.306)	(0.358)	(0.273)	(0.193)
Stage and Round Controls	Yes	Yes	Yes	Yes	Yes	Yes
Individual Controls	Yes	Yes	Yes	Yes	Yes	Yes
Observations	1640	420	420	400	400	1640
Subjects	164	84	84	80	80	164

Notes: Clustered standard errors in group level in parentheses. *** denote significance at the 0.01 levels.

**Table 6:** Relationship between declared and actual returns.

	(1)	(2)	(3)	(4)	(5)	(6)
	Pooled	B	Cp	Pooled	B	Cp
Declared_Amount_Returned	0.693 ***	0.660 ***	0.751 ***			
	(0.127)	(0.205)	(0.163)			
Declared_Amount_Returned ^2^	−0.011 *	−0.013	−0.013			
	(0.006)	(0.013)	(0.008)			
Declared_Proportion_Returned				0.751 ***	0.551 *	2.177 ***
				(0.185)	(0.287)	(0.551)
Declared_Proportion_Returned ^2^				−0.495 **	−0.269	−1.620 ***
				(0.207)	(0.363)	(0.410)
Constant	4.226	0.234	8.561 *	0.395 *	0.207	0.182
	(3.111)	(3.849)	(5.158)	(0.216)	(0.278)	(0.354)
Stage and Round Controls	Yes	Yes	Yes	Yes	Yes	Yes
Individual Controls	Yes	Yes	Yes	Yes	Yes	Yes
Observations	420	210	210	298	131	167
Subjects	84	77	80	80	64	75

Notes: Clustered standard errors in group level in parentheses. *, **, and *** denote significance at the 0.1, 0.05, and 0.01 levels, respectively.

**Table 7:** Effects of lagged returns on amount sent in the next-round.

	(1)	(2)	(3)	(4)	(5)	(6)
	Declared	Actual	Both	Gap	Loss_c	Loss_i
Cp	0.275	0.312	0.377	0.905 ***	0.312	0.476 *
	(0.256)	(0.246)	(0.251)	(0.241)	(0.246)	(0.245)
Ct	0.384	0.127	0.094	0.037	0.127	0.178
	(0.260)	(0.247)	(0.253)	(0.224)	(0.247)	(0.242)
Cp × Ct	0.558 *	−0.146	−0.169	0.249	−0.146	0.120
	(0.326)	(0.310)	(0.307)	(0.335)	(0.310)	(0.315)
L_AmountSent	0.523 ***	0.393 ***	0.418 ***	0.743 ***	0.630 ***	0.739 ***
	(0.094)	(0.063)	(0.082)	(0.041)	(0.042)	(0.041)
L_declare_return_received	0.109 ***		−0.026			
	(0.037)		(0.044)			
L_actual_return_received		0.237 ***	0.250 ***			
		(0.034)	(0.038)			
L_return_gap				−0.149 ***		
				(0.038)		
L_loss_c					−0.237 ***	
					(0.034)	
L_loss_i						−2.108 ***
						(0.265)
Constant	2.596 *	3.395 ***	3.359 ***	2.350 *	3.395 ***	3.122 ***
	(1.355)	(1.249)	(1.264)	(1.350)	(1.249)	(1.177)
Stage and Round Controls	Yes	Yes	Yes	Yes	Yes	Yes
Individual Controls	Yes	Yes	Yes	Yes	Yes	Yes
Observations	656	656	656	656	656	656
Subjects	82	82	82	82	82	82

Notes: Clustered standard errors in group level in parentheses. *, and *** denote significance at the 0.1 and 0.01 levels, respectively.

**Table 8:** Moderator effect.

	(1)	(2)
	Gender	Ambiguity
Cp	1.770 ***	1.536 ***
	(0.603)	(0.491)
Ct	0.740	0.649
	(0.683)	(0.513)
Cp × Ct	−0.419	0.389
	(0.728)	(0.618)
Gender	0.377	
	(0.754)	
Gender × Cp	−0.597	
	(0.819)	
Gender × Ct	−0.323	
	(0.993)	
Gender × Cp × Ct	2.710 **	
	(1.096)	
Ambiguity		0.394
		(1.169)
Ambiguity × Cp		−0.475
		(0.698)
Ambiguity × Ct		−0.285
		(1.404)
Ambiguity × Cp × Ct		2.614 **
		(1.318)
Stage and Round Controls	Yes	Yes
Individual Controls	Yes	Yes
Observations	820	820
Subjects	82	82

Notes: Clustered standard errors in group level in parentheses. **, and *** denote significance at the 0.05 and 0.01 levels, respectively.

**Table 9:** Summary of hypothesis tests.

Hypothesis	Prediction	Empirical Finding	Supported?
H1: Responder competition increases trust and realized trustworthiness	Coefficients on Competition (Cp) positive for Amount Sent, Actual Amount Returned, and Actual Proportion Returned	Cp is positive and significant across all three outcomes (Table 3, columns 1–6). Competition increases Amount Sent by 1.457 (*p* < 0.01), Actual Amount Returned by 2.776 (*p* < 0.01), and Actual Proportion Returned by 0.101 (*p* < 0.01).	Yes
H2: Contract enforcement strengthens the effect of competition	Competition × Contract (Cp × Ct) positive and significant for trust and trustworthiness	Cp × Ct is positive and significant for Amount Sent (1.003, *p* < 0.1), Actual Amount Returned (3.444, *p* < 0.01), and Actual Proportion Returned (0.120, *p* < 0.01) (Table 3). The main effect of Contract alone is insignificant for Amount Sent, confirming that enforcement amplifies rather than independently drives trust.	Yes
H3a: Competition increases declared returns	Coefficient on Cp positive for Declared Amount Returned and Declared Proportion Returned	Cp = 4.962 (*p* < 0.01) for Declared Amount Returned; Cp = 0.193 (*p* < 0.01) for Declared Proportion Returned (Table 4). Competition strongly raises both the level and proportion of declared returns.	Yes
H3b: Higher declarations increase probability of selection	Declared_Higher positive and significant when sender choice is possible	Declared_Higher = 0.392 (*p* < 0.01) in Cp and 0.816 (*p* < 0.01) in CpCt (Table 5, columns 3 and 5). The effect is insignificant in random-matching treatments (B and Ct), confirming that declarations matter for selection only when senders can choose.	Yes
H4a: Declared returns informative but partially credible without enforcement	Positive association between declared and actual returns, but with a gap (actual < declared)	Declared returns positively predict actual returns in B and Cp (Table 6). However, the concave relationship in Cp (negative squared term, −1.620, *p* < 0.01) and the graphical evidence (Figure 5) show that very high declarations are only partially credible, creating a declared-actual gap that is eliminated under contract enforcement.	Yes
H4b: Senders update trust from realized declaration credibility	Lagged actual return positive; lagged declared-actual gap negative on next-round Amount Sent	L.actual_return_received = 0.250 (*p* < 0.01); L.return_gap = −0.149 (*p* < 0.01) (Table 7, columns 3–4). Senders respond primarily to realized returns rather than declarations alone, and a larger gap reduces subsequent trust.	Yes

**Figure A1:**
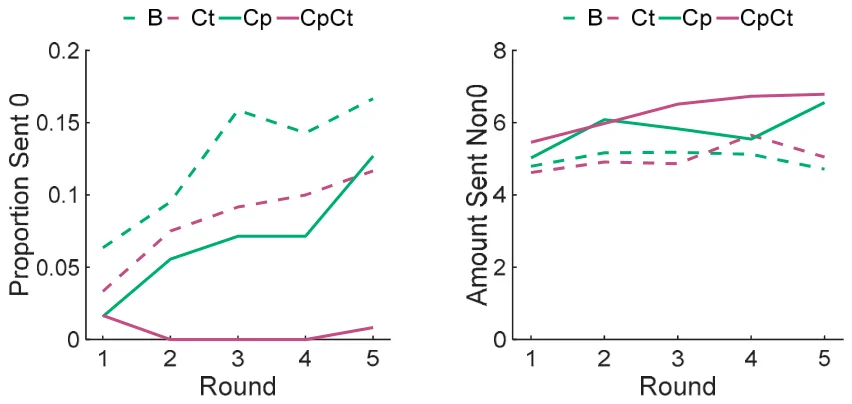
Dynamic patterns of extensive and intensive margins.

## Data Availability

The datasets generated and analyzed in the current study have been provided to the journal. They are not publicly available due to proprietary restrictions and their planned use in ongoing subsequent research, but are available from the corresponding author on reasonable request.

## Appendix A

### Appendix A.1. Decomposition of Treatment Effect: Extensive and Intensive Margins of Sent Amount

#### Appendix A.1.1. Extensive Margin

We decompose sender investment into the extensive margin and the intensive margin. The extensive margin captures whether a sender makes any positive investment, while the intensive margin captures the amount sent conditional on making a positive investment. This distinction is important because a higher average amount sent may arise either because more senders move away from zero investment, or because senders who already invest send larger amounts. Table A1 reports the descriptive statistics for these two margins.

**Table A1:** Descriptive statistics for extensive and intensive margins.

Outcome	B	Cp	Ct	CpCt
Positive-investment rate	0.624	0.795	0.750	0.985
Zero-investment rate	0.376	0.205	0.250	0.015
Conditional amount sent	4.992	5.749	4.995	6.301

The extensive-margin evidence is strong and consistent. The share of positive investments rises from 0.624 in B to 0.795 in Cp, to 0.750 in Ct, and to 0.985 in CpCt. Equivalently, the zero-investment share falls from 0.376 in B to 0.205 in Cp, to 0.250 in Ct, and to only 0.015 in CpCt. McNemar tests show that competition significantly increases the likelihood of making a positive investment in both institutional environments (B versus Cp, *p* < 0.001; Ct versus CpCt, *p* < 0.001). Two-sample tests of proportions confirm that contract enforcement also significantly affects the extensive margin: the difference between B and Ct (*p* = 0.006), while the difference between Cp and CpCt is highly significant (*p* < 0.001). Thus, a large part of the treatment effect on sender behavior works by drawing senders away from the corner solution of zero investment.

Figure A1 shows the dynamic trends. Spearman tests indicate clear dynamic patterns in zero-investment behavior. The incidence of zero investment increases significantly over rounds in the B (ρ = 0.222, *p* = 0.0012), the Cp (ρ = 0.250, *p* = 0.0002), and the Ct (ρ = 0.188, *p* = 0.0078). In contrast, no significant trend is observed in the CpCt (ρ = −0.058, *p* = 0.4360). Taken together, these results indicate that repeated interaction tends to push senders toward the corner solution of zero investment under most environments. Neither competition alone nor contract enforcement alone is sufficient to prevent this dynamic trust deterioration, whereas the joint presence of competition and contract enforcement effectively prevents this trend.

#### Appendix A.1.2. Intensive Margin

The intensive margin shows a more selective response to treatment factors. Conditional on making a positive investment, the average amount sent increases from 4.847 in B to 5.510 in Cp, remains essentially unchanged at 4.846 in Ct, and reaches 6.293 in CpCt. Competition significantly raises the conditional amount sent both without contract enforcement (Wilcoxon signed-rank tests: B versus Cp, *p* =0.0165) and with contract enforcement (Wilcoxon signed-rank tests: Ct versus CpCt, *p* < 0.001). By contrast, contract enforcement alone does not significantly affect the conditional amount sent in either environment (Wilcoxon rank-sum test: B versus Ct, *p* =0.7177; Cp versus CpCt, *p* = 0.1127). Therefore, the contract dimension mainly influences whether senders invest, whereas the competition dimension affects both participation and investment intensity.

Spearman tests in non-zero-investment amount and further support the findings above. Figure A1 shows no substantial divergence in investment intensity, while two treatments involving competition (Cp and CpCt) display mild upward trends over time. Consistent with this pattern, Spearman tests show no significant trend in B (ρ = −0.010, *p* = 0.9133) or Ct (ρ = 0.080, *p* = 0.3323). The Cp exhibits a marginally significant upward trend (ρ = 0.131, *p* = 0.0908), while the strongest increase appears in the CpCt (ρ = 0.151, *p* = 0.0341). These results suggest that conditional on positive investment, sent amount are broadly stable across treatments, although competition—particularly when combined with contract enforcement—gradually reinforces investment intensity over repeated interactions.

#### Appendix A.1.3. Treatment Effect of Extensive and Intensive Margins

Table A2 provides strong support for these two channels and is consistent with the descriptive pattern reported above. Columns (1) and (2) are the results of random-effect logit model regressions. The coefficient on Competition is positive and highly significant, indicating that competition increases the likelihood that the sender sends a positive amount. The coefficient on Contract is also positive, although its statistical significance is weaker in the logit specifications. More importantly, the interaction between Competition and Contract is positive and highly significant. These results indicate that competition and contract enforcement each independently increase the likelihood that a sender makes a positive investment, and their combination further strengthens this effect. Columns (3) and (4) show the margins effect, which is consistent with the results in columns (1) and (2).

**Table A2:** Treatment effects on extensive and intensive margins.

	(1)	(2)	(3)	(4)	(5)	(6)
	Send_Positive	Send_Positive	Send_Positive	Send_Positive	Amount Sent	Amount Sent
main						
Cp	1.252 ***	1.269 ***	0.180 ***	0.178 ***	0.759 **	0.754 **
	(0.397)	(0.397)	(0.034)	(0.031)	(0.332)	(0.333)
Ct	1.175 *	1.005 *	0.173 ***	0.154 ***	0.091	0.062
	(0.613)	(0.524)	(0.047)	(0.039)	(0.541)	(0.524)
Cp × Ct	2.961 ***	2.875 ***			0.540	0.539
	(0.821)	(0.815)			(0.452)	(0.453)
Constant	3.944 ***	3.814			4.710 ***	4.820 *
	(0.798)	(2.989)			(0.592)	(2.881)
Stage and Round Controls	Yes	Yes	Yes	Yes	Yes	Yes
Individual Controls	No	Yes	No	Yes	No	Yes
Observations	820	820	820	820	645	645
Subjects	82	82	82	82	82	82

Notes: Clustered standard errors in group level in parentheses. *, **, and *** denote significance at the 0.1, 0.05, and 0.01 levels, respectively.

Columns (5) and (6) examine the intensive margin by restricting the sample to observations with positive investment. The coefficient on Competition remains positive and statistically significant, suggesting that competition also increases the amount sent condition on positive investment. The estimated effect is about 0.75 units. By contrast, the coefficient on Contract is small and statistically insignificant, and the interaction between Competition and Contract is also not statistically significant in the conditional amount-sent regressions. This pattern suggests that contract enforcement affects sender behavior mainly by changing whether senders invest at all, rather than by substantially increasing the amount sent.

To sum up, competition affects both margins: it encourages more senders to make positive investments and also increases the conditional amount sent among positive investors. Contract enforcement, by contrast, primarily operates through the extensive margin. Its main role is to reduce fulfillment uncertainty and make competitive declarations credible, thereby preventing senders from choosing zero investment. This helps explain why the competition-and-contract treatment generates the largest amount sent: the combination sharply reduces the zero-investment corner and further enhances the intention to increase the amount sent due to competition between responders.

#### Appendix A.1.4. Decomposing the Increase in Amount Sent

The arithmetic decomposition used below is a decomposition based on the treatment $A_{t}=P_{t}\times M_{t},\;where\;t\;\in \;\{B,\;Cp,\;Ct,\;CpCt\}$, where $A_{t}$ denotes the unconditional mean amount sent in treatment t, $P_{t}$ is the positive-investment rate and $M_{t}$ is the conditional mean amount sent (Butler et al., 2016; Diederich et al., 2016; Huck et al., 2015; Diederich et al., 2022). For any ordered pair of treatments $t_{0}$ and $t_{1}$, the total change in average investment is:

$$\Delta A_{{t_{1}t}_{0}}=A_{t_{1}}-A_{t_{0}}$$

This change can be decomposed symmetrically into an extensive-margin component and an intensive-margin component:

$$\Delta A_{{t_{1}t}_{0}}=(P_{t_{1}}-P_{t_{0}})\times \frac{M_{t_{0}}+M_{t_{1}}}{2}+(M_{t_{1}}-M_{t_{0}})\times \frac{P_{t_{0}}+P_{t_{1}}}{2}.$$

The first term is the extensive-margin contribution: it captures the part of the change in average investment associated with the change in the positive-investment rate, evaluated at the average conditional amount sent across the two treatments. The second term is the intensive-margin contribution: it captures the part associated with the change in the conditional amount sent, evaluated at the average positive-investment rate across the two treatments. The two terms add up to the total change in mean amount sent.

Table A3 shows the results. The decomposition of average investment helps explain why the extensive margin is central. The increase from B to Cp can be decomposed into an extensive-margin contribution of 0.921 and an intensive-margin contribution of 0.536. Hence, about 63.2% of the increase from B to Cp comes from the reduction in zero investments. The contrast between B to Ct is even sharper: the increase is 0.632 in total, of which 0.630, or 99.7%, is attributable to the extensive margin. This implies that contract enforcement by itself primarily makes some previously inactive senders begin investing, but does not materially change how much active senders invest.

**Table A3:** Decomposition of extensive and intensive margins.

Comparison	Total Change	Extensive Contribution	Intensive Contribution	Extensive Share	Intensive Share
B vs. Cp	1.457	0.921	0.536	0.632	0.368
Ct vs. CpCt	2.460	1.327	1.133	0.540	0.460
B vs. Ct	0.632	0.630	0.002	0.997	0.003
Cp vs. CpCt	1.635	1.143	0.492	0.699	0.301
B vs. CpCt	3.093	2.040	1.053	0.660	0.340

The combined treatment produces the strongest overall response because it improves both margins simultaneously. From Cp to CpCt, the increase in Amount Sent is 1.635, with 69.9% of that increase attributable to the extensive margin and 30.1% to the intensive margin. From Ct to CpCt, the increase is 2.460, split more evenly between the extensive margin (54.0%) and the intensive margin (46.0%). Taking B as the baseline, the full combined-treatment effect is 3.092, of which 66.0% is explained by the extensive margin and 34.0% by the intensive margin. The decomposition indicates that the combination of competition and contract enforcement not only makes positive investment nearly universal, but also increases the amount sent by those who already participate.

Result A: Competition affects both margins: it encourages more senders to make positive investments and also increases the conditional amount sent among positive investors. Contract enforcement, by contrast, primarily operates through the extensive margin, which makes some previously inactive senders begin investing.

## Appendix B. Learning Effect of Trust

### Appendix B.1. Subsample Regression on Lagged Gap

Table A4 reports treatment-specific regressions for the lagged declared-actual return gap in the two non-contract settings, B and Cp. In both treatments, the lagged Amount Sent is positive and significant, showing persistence in sender investment. More importantly, the lagged return gap is negative and significant in both B (−0.197, *p* < 0.01) and Cp (−0.154, *p* < 0.01). This confirms that when declarations are not automatically enforced, senders reduce subsequent investment after observing a larger gap between what was declared and what was actually returned.

**Table A4:** Subsample regression on lagged actual and declared return gap.

	(1)	(2)
	B	Cp
L_AmountSent	0.632 ***	0.897 ***
	(0.072)	(0.053)
L_return_gap	−0.197 ***	−0.154 ***
	(0.050)	(0.050)
Constant	5.560 **	2.105
	(2.319)	(2.791)
Stage and Round Controls	Yes	Yes
Individual Controls	Yes	Yes
Observations	168	168
Subjects	42	42

Notes: Clustered standard errors in group level in parentheses. **, and *** denote significance at the 0.05 and 0.01 levels, respectively.

### Appendix B.2

Table A5 provides additional treatment-specific evidence using sender-side return-experience measures. Columns (1)–(4) use the continuous lagged gap between the sender’s previous Amount Sent and the actual return received. The coefficient is negative and significant in all four treatments, indicating that the larger the gap between what the sender invested and what the sender received back, the lower the sender’s next-round investment. In other words, when senders receive less realized reciprocity relative to their own prior investment, they subsequently reduce trust.

Columns (5)–(8) replace the continuous gap with a loss indicator. This indicator equals one when the sender’s previous Amount Sent exceeds the actual return received, and zero otherwise. The loss indicator is negative and significant in B, Cp, and CpCt, while the coefficient is negative but statistically insignificant in Ct. This indicates that facing a loss in the previous round leads senders to invest less in the following round. These results support the main dynamic-learning interpretation: senders update trust downward when previous investment outcomes reveal insufficient realized reciprocity or a net loss.

**Table A5:** Subsample regression on lagged amount sent and actual return gap.

	(1)	(2)	(3)	(4)	(5)	(6)	(7)	(8)
	B	Cp	Ct	CpCt	B	Cp	Ct	CpCt
L_stage_AmountSent	0.640 ***	0.701 ***	0.496 ***	0.510 ***	0.685 ***	0.782 ***	0.508 ***	0.637 ***
	(0.060)	(0.059)	(0.108)	(0.102)	(0.059)	(0.051)	(0.137)	(0.078)
L_gap_s_ar	−0.341 ***	−0.276 ***	−0.189 ***	−0.128 **				
	(0.075)	(0.055)	(0.060)	(0.055)				
L_loss_a					−2.551 ***	−2.433 ***	−0.588	−2.068 ***
					(0.394)	(0.436)	(0.505)	(0.709)
Constant	5.171 **	1.269	5.680 **	2.403	6.276 ***	2.908	5.146 **	1.669
	(2.215)	(2.219)	(2.221)	(2.608)	(2.410)	(2.345)	(2.467)	(2.118)
Stage and Round Controls	Yes	Yes	Yes	Yes	Yes	Yes	Yes	Yes
Individual Controls	Yes	Yes	Yes	Yes	Yes	Yes	Yes	Yes
Observations	168	168	160	160	168	168	160	160
Subjects	42	42	40	40	42	42	40	40

Notes: Clustered standard errors in group level in parentheses. **, and *** denote significance at the 0.05, and 0.01 levels, respectively.
